# Can we, should we, eradicate the meningococcus?

**DOI:** 10.1016/j.vaccine.2011.12.068

**Published:** 2012-05-30

**Authors:** Martin C.J. Maiden, Matthias Frosch

**Affiliations:** a Department of Zoology, University of Oxford, South Parks Road, Oxford OX1 3PS, United Kingdom; b Institut fuer Hygiene und Mikrobiologie, Universitaet Wuerzburg, Josef-Schneider-Strasse 2, 97080 Wuerzburg, Germany

**Keywords:** *Neisseria meningitidis*, Vaccines, Epidemiology, Population biology

## Abstract

The eradication of infectious agents is an attractive means of disease control that, to date, has been achieved for only one human pathogen, the smallpox virus. The introduction of vaccines against *Neisseria meningitidis* into immunisation schedules, and particularly the conjugate polysaccharide vaccines which can interrupt transmission, raises the question of whether disease caused by this obligate human bacterium can be controlled, eliminated, or even eradicated. The limited number of meningococcal serogroups, lack of an animal reservoir, and importance of meningococcal disease are considerations in favour of eradication; however, the commensal nature of most infections, the high diversity of meningococcal populations, and the lack of comprehensive vaccines are all factors that suggest that this is not feasible. Indeed, any such attempt might be harmful by perturbing the human microbiome and its interaction with the immune system. On balance, the control and possible elimination of disease caused by particular disease-associated meningococcal genotypes is a more achievable and worthwhile goal.

## Highlights

► New meningococcal vaccines are being developed and implemented. ► We examine the degree of meningococcal disease control achievable with vaccination. ► Population and evolutionary considerations are important in control of meningococci. ► Eradication of all meningococci is currently neither achievable nor desirable. ► Control of particular meningococci is an achievable end that can be attempted.

## 1 The control, eradication, and elimination of infectious diseases

Throughout history infectious diseases have emerged as a consequence of the ways that human populations have changed their ecology. Before the acceptance of the germ theory of disease, the capacity of human beings to react to these diseases was very limited, but over the last 120 years or so we have become increasingly able to anticipate the spread of diseases and make deliberate ecological interventions to prevent them or reduce their impact [1]. Whilst many of these interventions have been spectacularly successful and made urban living both possible and even pleasant, the ultimate goal of eradicating an infectious disease has been achieved in only one case, that of smallpox. The reasons for the success of this campaign, now over 30 years ago, are still instructive: small pox was antigenically stable; infection and immunisation both gave lifelong protection; there was no animal reservoir and no asymptomatic carrier state in humans; a safe universal vaccine that could be produced and delivered world-wide was available; and there was a strong political and public will to combat this terrible and debilitating disease [2]. The difficulties encountered by subsequent attempts to eradicate other diseases reflect the fact that none of them have met all of these criteria [3].

The Dahlem workshop defined a hierarchy of five levels of containing infectious diseases: control; elimination of disease; elimination of infections; eradication; and extinction (Table 1) [4]. As novel vaccines are developed against pathogens it is appropriate to examine the level in this hierarchy that can be achieved. There is a natural desire to employ these new products to eliminate or eradicate the disease in question. Here we will examine this question for *Neisseria meningitidis*, the meningococcus, in the light of the vaccines currently being developed and deployed against this encapsulated bacterium [5]. As the most effective of these vaccines target the asymptomatic carriage and transmission of meningococci among individuals [6], the question of whether elimination or eradication can be achieved arises. Clearly, the best way to prevent an infectious disease is to stop the circulation of the causative agent and indeed drive it to extinction: if the pathogen is not present it cannot cause pathology. In the case of the meningococcus, which is an important cause of septicaemia and meningitis world-wide [7], there are historical hints of a meningococcal disease-free world in that this very distinctive disease was not conclusively described before 1805 in Europe [8] and only towards the end of the 19th century in sub-Saharan Africa [9]. Is it possible to return to this desirable state? If this course is to be considered, it is necessary to examine its feasibility and consequences in the light of the biology of this intriguing organism.

## 2 Meningococcal biology

The meningococcus is only known to inhabit the human nasopharynx, if one discounts its occasional isolation from the human urogenital tract – the niche for its close relative the gonococcus [10]. It is asymptomatically carried in all human populations examined to date, albeit at variable prevalence [11,12]. Further, it has not been isolated from other animals and no known animal reservoir exists [10]. Carriage, which is rare in infants, increases with age and is episodic: an individual will acquire a particular meningococcus, carry that meningococcus for a period of time, which may range from days to years, and then clear the infection – remaining susceptible to infection by another meningococcus [13,14]. It is not known why some episodes of carriage develop into disease, especially as this is unproductive for the bacterium as invasion of the bloodstream, CSF, and meninges cannot lead to onward transmission [15]. Meningococcal disease should regarded as a dysfunctional relationship which harms the host and, ultimately, also the bacterium [16].

Some of the answers to the paradox of a commensal causing disease in a way that does not promote its own spread may lie in the extremely high diversity of this bacterium [16]. *N. meningitidis* possesses multiple mechanisms for generating antigenic variants by altering the levels of expression of multiple genes [17,18]. Presumably this aids interaction with a wide variety of human receptors for the purposes of colonisation and for the evasion of immune responses [19]. In addition to this intra-strain diversity, meningococci are highly diverse at the population level, with many thousands of distinct genotypes identified by various molecular typing methods, many generated by frequent genetic exchange among these highly transformable organisms [20]. This continual within and among-host evolution is likely to occasionally generate variants that are more likely to cause disease; however, mostly these are maladaptive and will not spread beyond the host in which they arise. One body of theory suggests that it is only mildly pathogenic variants that spread to cause large outbreaks, as they incur only a small cost for their pathogenicity [21]. In any case, it is likely: (i) that particular cell components have ambiguous rolls, promoting asymptomatic transmission but also increasing the likelihood of causing disease; and, (ii) that different circulating genotypes are a consequence of evolutionary forces that act to balance transmission efficiency against their likelihood to cause invasive disease [22].

In common with the great majority of bacteria that inhabit the nasopharynx, most meningococci present no risk to human health – a substantial proportion of meningococci possess no capsular locus [23], and only six of the 12 capsular serogroups are associated with disease, with five of these, serogroups A, B C, W and Y, responsible for most cases of invasive disease [24]. Multiple distinct genotypes exist which can be identified by multilocus sequence typing (MLST) as sequence types, which can be grouped into clonal complexes [25]. These are stable over decades and during global spread, but only a small number of them – the so-called ‘hyperinvasive lineages’ – cause most invasive disease [16]. The genetic factors responsible for the hyperinvasive phenotype are incompletely understood: although virtually all invasive meningococcal isolates have a polysaccharide capsule, and a number of other genes and gene products have been implicated in invasion [15]. The role of most of these is much more ambiguous, as none are found in all invasive meningococci, and many are shared with less invasive meningococci and other members of the genus *Neisseria* that do not cause invasive infections [26–28].

The meningococcus thus represents a common member of the microbiota of the human nasopharynx which rarely causes disease. Even in the case of the hyperinvasive meningococci, most episodes of carriage are asymptomatic [29]. It is likely that carriage of these organisms has some benefit to the host, even if this is only preventing other more pathogenic bacteria occupying the same niche. Carriage of the close relative of the meningococcus, the acapsulate *Neisseria lactamica*, for example, is very common in infants but invasive disease cause by this bacterium is extraordinarily rare [30]. Almost certainly the carriage of these organisms results in the development of an immune response and, as individuals age, they acquire immunity against invasion from carriage [31]. Hence, in planning public health interventions that target meningococci by affecting the carrier state, it is necessary to consider (i) which meningococci to target and (ii) the broader implications of removing meningococci from carriage, as this may perturb natural immunity.

### 2.1 Why would we want to eradicate the meningococcus?

From the perspective of the clinician, especially the paediatrician, the eradication of the meningococcus is a highly attractive concept [32]. Meningococcal disease is a sudden onset and very severe syndrome, principally affecting the very young, and an infected individual can deteriorate from being apparently perfectly healthy to presenting a medical emergency in a matter of a few hours. Even in countries with access to state-of-the-art medical facilities children still die when the race between diagnosis and treatment and bacterial growth in the blood stream and/or cerebro spinal fluid and is lost [33]. Individuals who survive frequently suffer debilitating sequelae, further magnifying the impact of this much-feared disease, even when disease rates are relatively low [34]. In resource poor settings, the impact of the disease is even greater, especially the meningitis belt of Africa, which experiences large-scale epidemic outbreaks of meningococcal meningitis [9]. These outbreaks represent the highest burden of meningococcal disease worldwide. They occur periodically, slightly more often than once a decade, over a period of 5–6 weeks in the dry season during the period of the trade wind, the Harmattan. In addition to causing tens of thousands of case and hundreds or thousands of deaths, these outbreaks are very disruptive, overwhelming healthcare systems for their duration [35].

### 2.2 What level of disease control is feasible?

On the balance of the evidence currently available, the eradication of the meningococcus *per se* is not desirable, even if it were achievable, which appears unlikely with current or foreseeable technology. As most infections with the meningococcus are harmless to the human host, deliberately removing a common component of the commensal microbiota could have consequences that are not easily anticipated, for example the exploitation of the vacated niche by other, more harmful, organisms leading to the increase similar or different pathologies. A further risk of targeting all meningococci indiscriminately is that this may well be only partially successful and could lead to the elimination of normally harmless meningococci, resulting in the paradoxical rise in disease as passive and active protection accorded to the host population by the carriage of these organisms is lost. Indiscriminate intervention in a system that we do not understand is unwise.

Public health interventions are more appropriately targeted to the control of the disease, rather than the eradication of the meningococcal population as a whole. This is a much more achievable goal, with fewer possible negative consequences. As the great majority of invasive meningococci are encapsulated, with most disease caused by a few serogroups, only bacteria expressing these capsular polysaccharides need be targeted. It is tempting to speculate that the pre-disease era was a time when fewer carried meningococci were encapsulated, at least with the disease-associated capsular serogroups. Tools for tackling meningococci that express four of the disease-associated seogroups (A, C, Y and W) are to hand in the form of protein-conjugate polysaccharide vaccines [5]. At least in the case of the meningococcal C polysaccharide conjugate (MCC) vaccines, immunisation of the population in which transmission is occurring can disrupt transmission to the extent that the circulation of potentially invasive organisms can be reduced to a very low level, if not completely eradicated [36,37]. In a number of countries this has been achieved for serogroup C meningococci, with little convincing evidence of the replacement of these organisms with other harmful meningococci. The goal would be to eliminate serogroup A, B, C, W, Y, and perhaps X capsules: more specifically this means removing from the meningococcal population the Region A variants of the *cps* genome region which encode the synthesis genes for these serogroups [38].

### 2.3 A strategy for the control or elimination of disease-associated meningococci

A three-phase programme for the control or elimination of invasive meningococci can be envisaged:

Phase I would target serogroup A and serogroup C meningococci at the global level. Effective conjugate vaccines exist against these organisms, including the recently introduced MenAfriVac vaccine [39], developed to be affordable in sub-Saharan countries [40].
Phase II would target serogroup Y, W and X meningococci with affordable conjugate vaccines against these organisms. Vaccines against serogroups Y and W exist [41], and it is likely that a serogoup X vaccine could be developed, although the disease burden of this serogroup remains limited [42]. There may be some cross-protection against strains expressing serogroup X by protein based vaccines developed against serogroup B meningococci. The ideal solution would be a five-valent conjugate polysaccharide vaccine (A, C, W, X, Y), which was effective against these serogroups and in both disease and carriage.
Phase III would target serogroup B, but this is much more problematic. There is no conjugate polysaccharide vaccine available against this serogroup, and little prospect of one being developed [43]. This phase, which would be important to ensure that the current burden of disease is not simply replaced by an increase in serogroup B disease, therefore relies on a successful research and development program to develop a broadly protective ‘serogroup B substitute’ vaccine.

## 3 Can this strategy be realised?

Phases I and II are feasible with current technology, if challenging from a logistical point of view. Indeed, in one of the most exciting developments in the history of meningococcal disease control, the rollout of the MenAfriVac conjugate serogroup A polysaccharide vaccine presents the prospect of the end of epidemic group A meningococcal disease in sub-Saharan Africa [35]. The goal of the Meningitis Vaccine Project (MVP) was the sustainable introduction of a serogroup A conjugate polysaccharide vaccine, with the vaccine priced a less 1US$ per dose, a goal that was achieved by a novel North–South partnership of technology transfer and manufacturing capacity [40]. Other factors aiding the elimination of serogroup A meningococci is their relative lack of genetic diversity and geographical distribution. Virtually all cases of serogroup A disease are caused by one of three clonal complexes, ST-1 complex and the closely related ST-4 and ST-5 clonal complexes [44]. This is different from sialic acid-containing serogroups B, C, W and Y which are found in numerous genetically divergent clonal complexes. Similarly, whilst the sialic acid capsules are globally distributed, much of the serogroup A disease is in Africa and Asia [9,44,45], with certain regions currently experiencing little or no serogroup A disease [16]. After Africa, therefore a major effort of introduction of MenAfriVac into Asia could see a dramatic reduction in the disease burden of group A meningococci, perhaps resulting in the elimination of this disease globally and perhaps even the eradication of group A meningococci [35].

Similar arguments can be made for the MCC vaccines, which have achieved virtual eradication of serogroup C meningococcal disease in a number of countries where it has been introduced [46]. It should be noted here that it is more accurate to say that serogroup C ST-11 complex meningococci, which express their capsules at high rates, have been eradicated [37]. It is possible that other genotypes which express the capsule at lower rates, and are consequently less susceptible MCC vaccines, could act as a reservoir for the genes encoding the serogroup C capsule, making its eradication difficult. A further problem is that meningococci that express this capsule are globally distributed [16], including in countries that have low incidence rates of disease, which might be resistant to the universal introduction of a vaccine against an organism which represents only a modest threat to their public health – evidence for this is the patchy introduction of this vaccine in European counties. Those countries which have immunised children and young adults with MCC vaccines, such as the United Kingdom and the Netherlands, have exhibited the most dramatic reductions in serogroup C disease [36,47].

Compared with Phase I, Phase II presents a number of uncertainties. Serogroups W and, particularly, Y are less common causes of disease and are commonly carried. In addition they are found in a range of clonal complexes, a number of which very rarely cause disease and their rates of capsule expression during carriage are lower, ranging from 28 to 70%, depending on the clonal complex [29,48]. Experience from the UK MCC introduction suggests that it was the high rate of capsule expression in carriage, combined with genetic uniformity of the ST-11 complex serogroup C meningococci, which resulted in the high impact of the vaccine [37]. Extrapolating this success to other serogroups, especially Y and W may well be optimistic. More worryingly, the apparently very low invasive potential of serogroup Y ST-22 complex meningococci [29], suggests that their elimination may be detrimental to disease control, at least whilst other more invasive meningococci are still circulating. Very high rates of serogroup Y carriage have been reported and, whilst these have been associated with increases in rates of serogroup Y disease, these remain very low compared with the disease rates that occur during periods of elevated transmission of hyperinvasive serogroup B and C meningococci [29]. It is at least possible the serogroup Y organisms prevent disease by excluding more harmful organisms and attempting their elimination must take this into account. Further, the low levels of capsule expression of some clonal complexes associated with serogroup Y during carriage [48] may render their elimination impossible with current approaches.

Phase III – the elimination of serogroup B meningococci, is by far the most challenging goal both from the perspective of the tools currently available and the biology of the meningococcus [5,43]. The first, and by far the most serious problem, is that the development of a conjugate vaccine against the serogroup B polysaccharide is precluded by a combination of the poor immunogenicity of this polysaccharide and safety concerns, as it is identical to a host antigen NCAM, which decorates foetal neural tissues [49]. Further, a number of quite different clonal complexes that express serogroup B have been associated with disease and over the last decades several have emerged and spread globally in succession [16]. To date, all of the ‘serogroup B substitute’ vaccines that have been implemented have been based on the proteins expressed on the surface of a particular meningococcal strain [50–52]. These provide protection against disease caused by that strain and close relatives, *i.e.* members of the same clonal complex that express similar antigenic variants, but not against others [53]. More sophisticated formulations that increase the coverage against serogroup B meningococci are being aggressively developed [54,55]. It will be interesting to learn whether these vaccines will be able to interrupt transmission to the extent that eradication or elimination would be possible.

## 4 Conclusion

The eradication of all carried meningococci is almost certainly neither achievable nor desirable: the control, elimination, or eradication of particular invasive meningococci is a more realistic goal, given that a limited number of clonal complexes and serogroups cause most meningococcal disease. However, even this goal is likely to be difficult and requires more research and great political will to achieve. In terms of practicability and desirability, the MenaAfriVac vaccine and its introduction indicates how epidemic serogroup A disease can potentially be eliminated or eradicated. The other serogroups are more problematic. Serogroup C is the next most likely candidate, although the biology and logistics are less favourable than for serogroup A. Serogroups Y and W could be targeted, but the cost benefit of this is less clear at the current time. In principle a serogroup X vaccine could be developed, but whether the disease burden is sufficient to warrant its introduction at a scale sufficient for eradication of group X meningococci is doubtful. Finally, and perhaps most problematically, no tools currently exist for controlling serogroup B meningococci *per se*, and although it may be possible to develop vaccines that target particular or even most serogroup B-associated clonal complexes, thereby substantially reducing disease burden, eradicating all group B meningococci from carriage globally is unlikely to be feasible. Consequently, a world free of invasive meningococci remains an alluring but still distant prospect, although a world with appreciably less meningococcal disease is an achievable and worthwhile goal in the immediate future.

## Conflict of interest statement

MCJM: named inventor on patent applications in the area of meningococcal vaccines and occasional external consultancy (Novartis, GSK, Sanofi Pasteur and Wyeth). MF: Declares no potential conflict of interest.

## Figures and Tables

**Table 1:** The Dahlem workshop definitions of public health interventions and examples.

Hierarchy of Intervention	Definition	Example
Control	The reduction of disease incidence, prevalence, morbidity or mortality to a locally acceptable level as a result of deliberate efforts; continued intervention measures are required to maintain the reduction	Diarrhoeal diseases
Elimination of disease	Reduction to zero of the incidence of a specified disease in a defined geographical area as a result of deliberate efforts; continued intervention methods are required	Neonatal tetanus
Elimination of infections	Reduction to zero of the incidence of infection caused by a specific agent in a defined geographical area as a result of deliberate efforts; continued measures to prevent reestablishment of transmission are required	Measles, Poliomyelitis
Eradication	Permanent reduction to zero of the worldwide incidence of infection caused by a specific agent as a result of deliberate efforts; intervention measures are no longer needed	Smallpox
Extinction	The specific infectious agent no longer exists in nature or the laboratory	None
